
JOURNAL INFORMATION
==============================
NLM Title Abbreviation: Buffalo Med J Mon Rev Med Surg Sci
NLM Journal ID: 0004276

The Buffalo Medical Journal and Monthly Review of Medical and Surgical Science

Publisher: Biomedical Journal Digitization Project

ARTICLE INFORMATION
==============================
PMCID: PMC8932455
PMID: 35376952
Article version: 1
Subjects: Eclectic Department

Anæsthetics

Print publication date: 1858 Dec
Volume: 14
Issue: 7
First page: 434
Last page: 435
License: This work is free of known copyright restrictions. For more information, please see PMC Back Issue Digitization.
License URL: https://creativecommons.org/publicdomain/mark/1.0/

==============================

her only hope, This she did, for a time, but soon her shrieks and groans became heart-rending. Her husband and son now rushed into the room, and commanded me to desist from further attempts to relieve the patient; which I did only after at least two quarts had been injected. The friends evidently looked upon me as a personification of brutality. Though conscious of having done my duty, I felt confident I had lost the confidence of the many present, and that I had better have let the patient died, at least without the last effort for relief.

About an hour after this, to my great joy, the bowels moved freely and repeatedly, to the no inconsiderable relief of the patient. From this time on, the only laxative required was a little yeast. The patient, however, was not destined to recover. She died about two weeks later, seemingly from an inability to rally from the extreme prostration. Peritoneal inflammation was, doubtless, the cause of death, which, in a person of less years, and under more favorable circumstances, might have been cured.

I can not avoid the conviction, that if the copious injections, with a view of forcing the obstruction, had been resorted to earlier, the result might have been different.

Frewsburg, Chatauque Co., N. Y.

---

*Anæsthetics.*—We quote the following article from the Lancet for October 16, 1858, and we have no doubt that all it asserts is true. Indeed, we have looked over the late Dr. Snow's work with much interest, and can heartily join in the recommendation of the Lancet, to give this "valuable monograph" a "thoughtful perusal." Notwithstanding all that is said, however, even by the highest authority, we believe that the time is not far distant when chloroform will be abandoned as an anæsthetic agent. We again ask, why use an article of such uncontrollable power, when we have an equally reliable one in ether, and one which is entirely safe? It is notorious, that under the best conditions, and in the most careful hands, chloroform is a dangerous weapon. It is only lately that Mr. Erichsen, high surgical authority, has stated both pubilcly and privately, that he uses chloroform as he wonld take an *express train*, ignoring, or risking, the dangers of extra velocity. Now, is it not better to travel on the anæsthetic track a little more slowly, and be safe in our journey? Mr. Erichsen himself intimates as much as this; and the evidence of every day only confirms us in the opinions we have expressed.

"The frequent recurrences of death from the administration of chloroform during the last few weeks has induced a feeling of uneasiness and distrust of this agent both in the profession and out of it. It is very certain, however, that a great part of this feeling might very advantageously be transferred to the mode of administration which is still in vogue. It must not be forgotten that, in all these cases, the chloroform was administered loosely on a handkerchief, and in more than one instance, as has been proved, it was of a thickly-woven texture, which did not allow of a free passage of air. This mode of administration is, we think, most unjustifiable. The experiments of the late Dr. Snow conclusively showed the necessity of carefully regulating the proportion of vapor in the air inspired. We strongly recommend to thoughtful perusal the valuable monograph on the subject of Anæsthesia which he has bequeathed to the profession. No one can rise from reading this valuable digest of a wide experience and the observation of ten years of scientific and practical labor, without a feeling of regret that so much carelessness should still prevail in the administration of this most potent vapor, and a sense of the necessity for a more extended instruction in the principles of anæsthetization. We commend the following sentences to the very careful consideration of surgeons:—

"The great point to be observed in causing insensibility by any narcotic vapor, is to present to the patient such a mixture of vapor and air as will produce its effects gradually, and enable the medical man to stop at the right moment. Insensibility is not caused so much by giving a dose as by performing a process. Nature supplies but one mixture of diluted oxygen, from which each creature draws as much as it requires; and so, in causing narcotism by inhalation, if a proper mixture of air and vapor is supplied, each patient will gradually inhale the requisite quantity of the latter to cause insensibility, according to his size and strength. It is, indeed, desirable to vary the proportions of vapor and air, but rather according to the purpose one has in view, whether medicinal, obstetric or surgical, than on account of the age or strength of the patient; for the respiratory process bears such a relation to the latter circumstances, as to cause each person to draw his own proper dose from a similar atmosphere in a suitable time.'

"The proportion of chloroform most suitable to produce insensibility, Dr. Snow found to be about four cubic inches of vapor, or rather more than five grains of chloroform to one hundred cubic inches of air. With a properly-arranged inhaler, it is easily possible to supply the vapor in this fixed position. This simple precaution would rob chloroform of nearly all its terrors and its dangers. It is a very surprising consideration, that while the niceties of surgical manipulation are invaribly attended to with the utmost care, the production of anæsthesia is very frequently left in the hands of quite inexperienced persons. It is sad to see the issues of life and death treated with indifference; it would be yet more so, if we were not well aware that the fault is one of thoughtlessness, and that those who have fallen into it will probably shrink from a similar error now that it has been pointed out so forcibly by recent events."—*Boston Med. and Surg. Jouraal.*

---

*Warning to Invalids Visiting the Island of Cuba.*—[We beg leave to present our readers the following extract from a letter received from an esteemed correspondent in Havana. It speaks for itself, and all medical men who send patients to Cuba for relief, should be careful to make every effort to secure them from the imposition pointed out.—Eds.]

If you like to take the trouble to give notice to your readers, that such of their patients and other invalids as come to Havana shall be counseled in respect to localities best suited to their conditions, or if so ill as to require private lodgings, or residences separate from the crowd and discomfort of hotels, it will give me much satisfaction to be of service to them in any way that may be in my power, and to carry out every judicious plan of treatment that may be recommended by their physicians.
